# Serum Proteomic Markers in Patients with Systemic Sclerosis in Relation to Silica Exposure

**DOI:** 10.3390/jcm14062019

**Published:** 2025-03-16

**Authors:** Mayka Freire, Bernardo Sopeña, Susana Bravo, Carlos Spuch, Ana Argibay, Melania Estévez, Carmen Pena, Martín Naya, Adela Lama, Arturo González-Quintela

**Affiliations:** 1 Unidad de Enfermedades Sistémicas e Inmunopatología, Servicio de Medicina Interna, Hospital Clínico de Santiago de Compostela, 15706 Santiago de Compostela, Spain; maria.del.carmen.freire.dapena@sergas.es (M.F.); martin.naya.rosato@sergas.es (M.N.); adela.lama.lopez@sergas.es (A.L.); 2 Laboratorio de Proteómica, Instituto de Investigación Sanitaria de Santiago, 15706 Santiago de Compostela, Spain; sbbravo@gmail.com (S.B.); carmen.pena.pena@sergas.es (C.P.); 3 Translational Neuroscience Group, Galicia Sur Health Research Institute (IIS Galicia Sur), SERGAS-UVIGO (Servizo Galego de Saúde-Universidade de Vigo), 36312 Vigo, Spain; carlos.spuch@iisgaliciasur.es; 4 CIBERSAM (Network Biomedical Research Center on Mental Health), 28029 Madrid, Spain; 5 Red de Investigación de Atención Primaria en Adicciones (RIAPAD), 08003 Barcelona, Spain; 6 Unidad de Enfermedades Autoinmunes Sistémicas y Trombosis, Servicio de Medicina Interna, Complejo Hospitalario Universitario de Vigo, 36312 Vigo, Spain; 7 Departamento de Psiquiatría, Radiología, Salud Pública, Enfermería y Medicina, Universidad de Santiago de Compostela, 15706 Santiago de Compostela, Spain

**Keywords:** Systemic sclerosis, scleroderma, silica, etiopathogenesis, proteomics

## Abstract

**Background:** Systemic sclerosis (SSc) is a multisystem autoimmune disease characterised by fibrosis, vasculopathy, and immune dysfunction. Silica exposure has been associated with a more aggressive phenotype of the disease, including diffuse cutaneous involvement and interstitial lung disease. This study aims to identify proteomic differences between SSc patients exposed to silica and those not exposed to silica. **Methods:** An observational study of 32 SSc patients (11 silica-exposed and 21 non-exposed) was performed, with occupational history and quantitative proteomic analysis using SWATH-MS mass spectrometry. Differentially expressed proteins were analysed, and functional pathway enrichment was performed. **Results:** Eight proteins showed significant differences between groups, all with reduced levels in silica-exposed patients: adiponectin, immunoglobulins (IGLV3-19, IGLV2-18), complement C2, alpha-2-macroglobulin, vitronectin, cytoplasmic actin 2, and pigment epithelium-derived factor. Alterations in pathways related to fibrinolysis, complement activation, and inflammation were highlighted, suggesting that silica exposure may influence the pathogenesis of SSc and worsen its clinical course. **Conclusions:** This study supports the hypothesis that silica exposure is not only a triggering factor for SSc, but is also modulating its progression through inflammatory, procoagulant, and fibrotic pathways. The identification of proteomic biomarkers could contribute to the phenotypic classification of patients and the development of personalised therapies. Future studies should expand the cohort and further investigate the functional mechanisms of these proteins in SSc.

## 1. Introduction

Systemic sclerosis (SSc) is a systemic autoimmune disease that is much more common in women (ratio 9:1), with an estimated prevalence in our region’s population of 0.82/100,000 [1]. Its prognosis can vary widely due to the high variability of its clinical expression, with an overall 10-year survival rate of 65–92% [2].

SSc is a complex disease in which immunological, vascular, and fibrotic factors interact. The pathogenesis of SSc is based on three fundamental pillars: autoantibody production, endothelial damage, and fibrosis due to excessive collagen deposition. As a result of these mechanisms, the function of virtually any organ can be altered, with the most commonly affected organs being the skin, gastrointestinal tract, lungs, heart, and kidneys [3].

Although the cause of SSc is unknown, several hormonal (17β-estradiol and prolactin), genetic (some genes associated with the X chromosome, HLA, and non-HLA genetic regions), and environmental (exposure to toxins such as silica dust, organic solvents, or vinyl chloride) factors have been described [4,5,6,7]. Of all the toxicants involved, silica is the most common in most series. Furthermore, a higher proportion of diffuse skin disease, interstitial lung disease, and mortality have been described in patients with SSc and a history of exposure to silica [7], suggesting that the toxin may not only act as a triggering mechanism for the disease but may also condition its phenotypic expression by inducing the activation of a specific pathogenic pathway.

**Figure 1:**
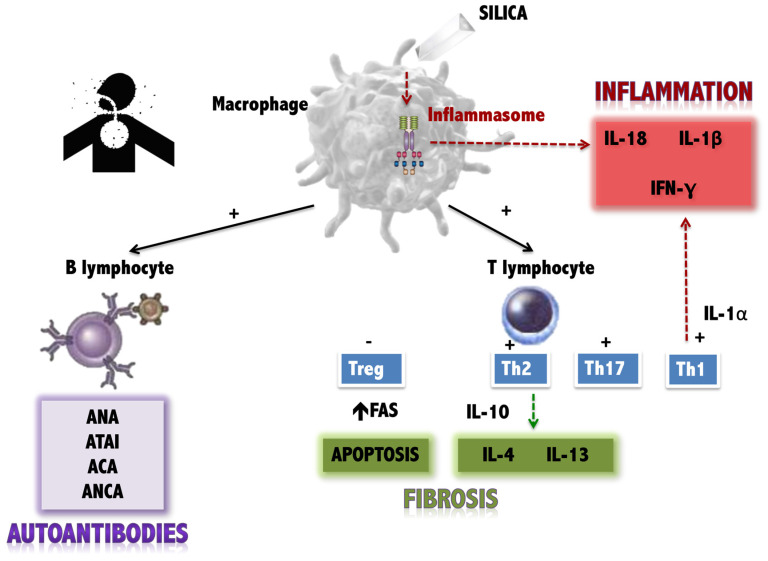
Current hypotheses about the effects of silica on the immune system. Antigen-presenting cells (such as the macrophage) are stimulated by silica, producing three types of effects on the immune system: (1) Stimulation of the inflammasome, with the production of pro-inflammatory cytokines (IL-18, IL-1beta). (2) Stimulation of T lymphocytes (both the Th1 response, also pro-inflammatory, and the Th2 response and attenuation of regulatory T lymphocytes, which favour fibrosis. (3) Stimulation of the production of autoantibodies by B lymphocytes (own elaboration, based on data collected in [8,9,10].

There are several hypotheses about the pathogenic mechanisms underlying silica exposure and the onset of autoimmune phenomena (Figure 1). However, little is known about the molecular mechanisms that trigger SSc in genetically predisposed individuals following exposure to the toxin.

Recently, Chairta et al. compiled and thoroughly analysed the candidate biomarkers in SSc discovered by mass spectrometry, collected from the 25 full-text studies published to date. This study showed that many proteins with different functions are involved in the pathogenesis of SSc, confirming the heterogeneity of the disease, as the reported deregulated proteins are involved in approximately 240 different pathogenic pathways. Therefore, we can say that different pathways and molecules are involved in different stages of SSc pathogenesis and different SSc subtypes [11]. Continued research into its molecular mechanisms is crucial to developing more effective therapies and improving the quality of life of patients.

The main objective of this work is to identify molecular patterns through proteomic analyses in a cohort of patients with SSc that will allow us to recognise a differentiating molecular signature between patients exposed and not exposed to silica dust. Identifying the predominant molecular pathways in the subset of exposed patients will allow hypotheses to be generated about the initial mechanisms of damage.

## 2. Material and Methods

### 2.1. Study Design

This observational study was conducted in patients with SSc who attended a routine follow-up consultation at the Systemic Autoimmune Diseases Unit in April 2018. All patients were diagnosed with SSc according to the ACR/EULAR 2013 criteria [12]. A work–life survey was performed, and exposure risk was assessed according to the specific recommendations for silicosis health surveillance of the Interterritorial Council of the National Health System of Spain [13]. Agriculture was also included among the occupations at risk of exposure to silica because digging in agriculture increases airborne silica exposure in farmworkers, as confirmed by lung autopsies showing higher mineral particle concentrations in agricultural workers [14,15]. furthermore, in our geographical environment, it is an activity carried out manually (digging with a hoe) on siliceous soil [16].

The inclusion and exclusion criteria were as follows:

- Inclusion criteria: patients exposed to silica dust were selected as cases (si-SSc) and patients not exposed as controls (nosi-SSc).
- The exclusion criteria were failure to provide written informed consent, comorbidity with another serious diagnosis or concomitant disease that could affect the results, pregnancy or lactation in women, or being a minor (<18 years).

### 2.2. Data Collection

- Working Life Epidemiological Survey.

Each patient was interviewed, and all the jobs they had in their life were recorded.

- Review of clinical and analytical data.

The computerised electronic history of each selected subject was accessed, and the most relevant clinical and analytical data were extracted.

### 2.3. Quantitative Proteomic Studies by Sequential Window Acquisition of All Theoretical Mass Spectrometry (SWATH MS) Method

- Protein Digestion.

The protein extract was loaded on a 10% SDS-PAGE (sodium dodecyl sulphate polyacrylamide gel electrophoresis) gel to the whole protein concentration. The gel was stained, and the band was exscinded and submitted to in-gel tryptic digestion, as described previously by our group [17,18], Peptides were extracted by carrying out three 20 min incubations in 40 μL of 60% ACN (acetonitrile) dissolved in 0.5% HCOOH (formic Acid), and then pooled, concentrated (SpeedVac, Thermo Fisher Scientific, Madrid, Spain), and stored at −2 °C.

- Mass Spectrometric Analysis by Sequential Window Acquisition of All Theoretical Mass Spectra (SWATH MS).

SWATH MS acquisition was performed on a TripleTOF^®^ 6600 LC-MS/MS (liquid chromatography couplet to mas spectrometry) system (AB Sciex LLC, Framingham, MA, USA). To build the SWATH library, a pool of each condition was analysed by a shotgun data-dependent acquisition (DDA) approach by micro-LC-MS/MS, an analysis already described by our group [19,20,21,22,23,24,25]. After protein identification using ProteinPilot, the ion density found in the DDA runs was used to create the MSMS SWATH method, adjusting the 100 SWATH windows to this ion density. Therefore, all individual samples were analysed using a data-independent acquisition method. The targeted data extraction from the SWATH MS runs was performed by PeakView v.2.2 (AB Sciex LLC, Framingham, MA, USA) using the SWATH MS Acquisition MicroApp v.2.0 (AB Sciex LLC, Framingham, MA, USA), and the data were processed using the spectral library created from DDA. SWATH MS quantization was attempted for all proteins in the ion library, but only those that have 10 peptides and 7 transitions per peptide were used for protein quantization.

The integrated peak areas were processed by MarkerView software version v.1.3.1 (AB Sciex LLC, Framingham, MA, USA) for a data-independent method for relative quantitative analysis. To control for possible uneven sample loss across the different samples during the sample preparation process, we performed most likely ratio normalisation [26]. Unsupervised multivariate statistical analysis using PCA (principal component analysis) was performed to compare the data across the samples. A Student’s *t*-test analysis on the averaged area sums of all the transitions derived for each protein in every sample will indicate how well each variable distinguishes between the two groups, reported as a *p*-value. For each library, its set of differentially expressed proteins (*p*-value < 0.05) with a FCh > 2 or < 0.5 was selected.

- Functional Enrichment and Interaction Network Analysis:

We performed functional enrichment and interaction network proteomes using STRING: functional protein association networks, https://string-db.org/ (accessed on 8 January 2025) (free access at https://string-db.org). We conducted a pathway analysis using Reactome, https://reactome.org (accessed on 8 January 2025) (https://reactome.org). Volcano plots were performed using GraphPad Prism v.9.0.0 (GraphPad Software, San Diego, CA, USA).

### 2.4. Ethical Aspects

The study complies with the Declaration of Helsinki. The Ethical Committee from Pontevedra-Vigo-Ourense has approved (12 June 2017) the research protocol with the number 2017/303. Written informed consent has been obtained from the subjects.

## 3. Results

### 3.1. Selected Patients

Thirty-two patients with SSc were included in the study. Of these, 11 had a history of occupational silica exposure (si-SSc group). A further 21 patients had not been exposed (nosi-SSc group).

### 3.2. Occupational Exposure Study

Table 1 shows the details of the occupations of all patients. The most common occupation in the si-SSc group was agriculture (three patients, 27%), followed by marble working, stonemasonry, and foundry (three patients, 18% each); one patient worked in a paper mill and later in the manufacturing of dental prostheses, both occupations with a risk of exposure to silica; and one last patient worked in a stonemasonry workshop.

### 3.3. Proteins with Statistically Significant Differences Between Exposed and Non-Exposed Groups

**Figure 2:**
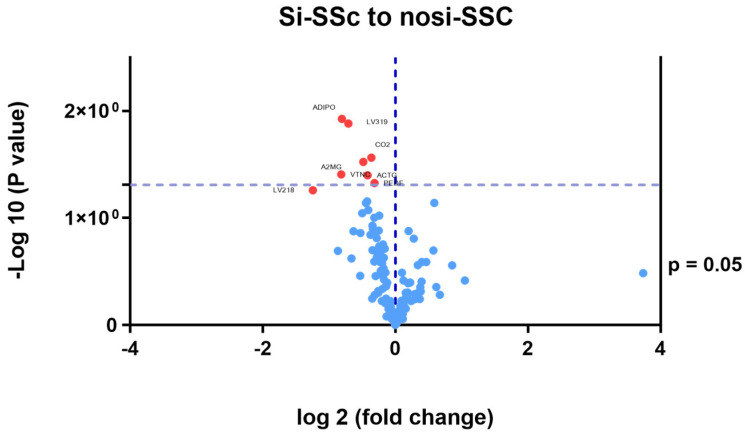
Differential proteins between si-SSc and nosi-SSc groups. SSc; systemic sclerosis; Si-SSc: systemic sclerosis patients with silica exposure; nosi-SSc: systemic sclerosis patients without silica exposure; Adipo: adiponectin; LV319: immunoglobulin lambda variable 3–19; CO2: complement C2; A2MG: alfa 2-macroglobulin; VTNC: vitronectin; ACTG: cytoplasmic actin 2; PED: pigment epithelium-derived factor; LV218: immunoglobulin lambda variable 2–18.

**Figure 3:**
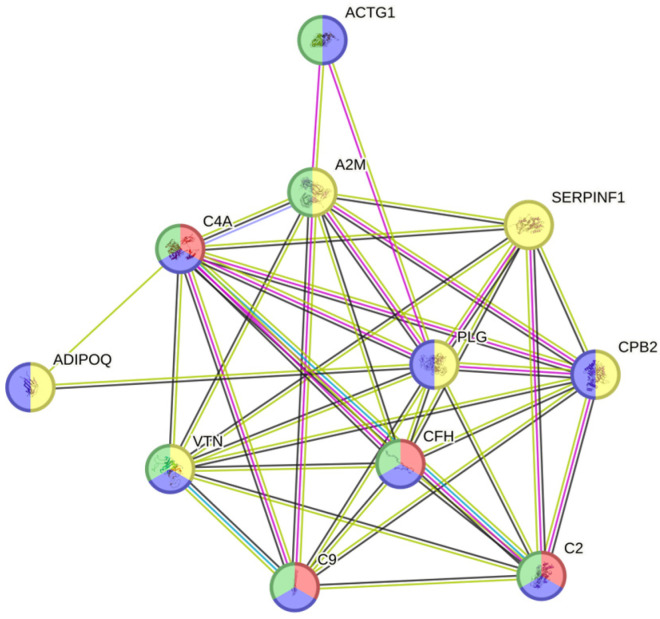
Protein Interaction Network. ADIPOQ: adiponectin; ACTG1: actin, gamma 1; PLG: plasminogen; C9: complement component 9; C2: complement component 2; C4A: complement C4-A; SERPINF1: Serpin Family F Member 1; CPB2: Carboxypeptidase B2; VTN: vitronectin; A2M: alpha-2-macroglobulin; CFH: Complement Factor H.

In the comparative study of the proteomic pattern between the si-SSc and nosi-SSc groups, statistically significant differences were found in eight proteins, all downregulated. (Table 2 and Figure 2): Adiponectin (log fold change −0.24), immunoglobulin lambda variable 3–19 (log fold change −0.21), immunoglobulin lambda variable 2–18 (log fold change −0.10), complement C2 (log fold change −0, 14), alpha-2-macroglobulin (log fold change −0.24), vitronectin (log fold change −0.12), actin, cytoplasmic 2 (log fold change −0.09), and pigment epithelium-derived factor (log fold change −0.37). A2M, PLG, and SERPINF1 stand out as hubs, indicating that they are essential in multiple processes related to the regulation of proteolysis, stress response, coagulation, and fibrinolysis (Figure 3). One part of the functional network is focused on coagulation and fibrinolysis (e.g., PLG, SERPINF1, CPB2), and another part is involved in the immune response (e.g., C4A, C9, CFH). The overlap between these domains suggests interrelated processes such as inflammation, tissue repair, and haemostatic regulation.

**Figure 4:**
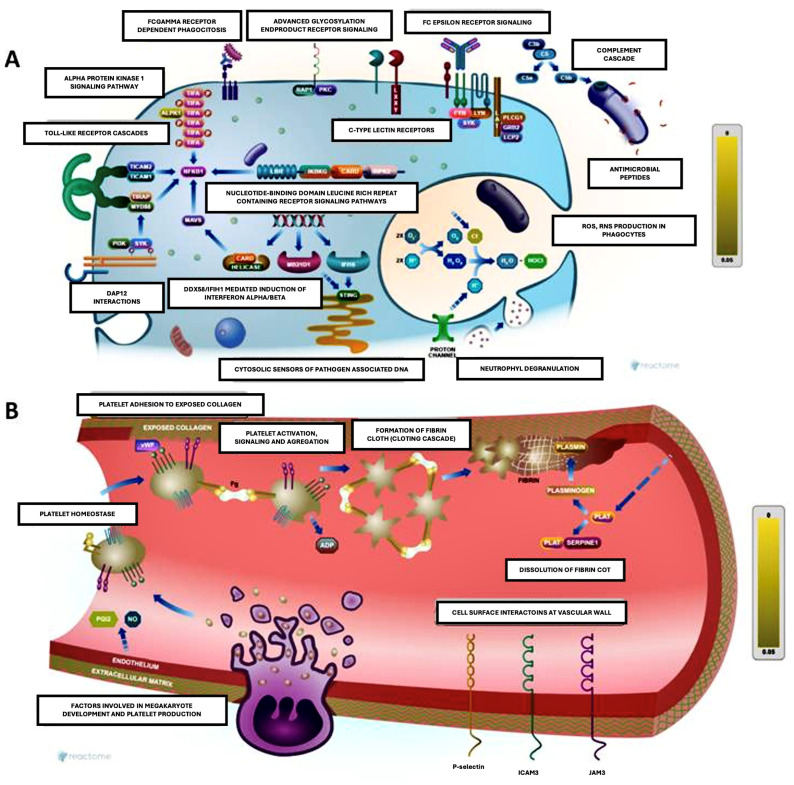
Figure made with Reactome Pathway Browser. Both schemes integrate immune and inflammatory processes in defence and repair contexts, highlighting the interconnection between immunity and coagulation. (**A**): Immune activation and molecular recognition (left). This scheme shows immune activation pathways, including pattern recognition receptors (PRRs), which detect danger or damage signals (PAMPs and DAMPs). Some key points include the following: toll-like receptors (TLRs) to detect bacterial components such as lipopolysaccharide (LPS) and activate intracellular signalling, resulting in the production of inflammatory cytokines; Fc receptors to mediate the response to immune complexes (antibodies bound to antigens), activating phagocytes and other immune cells; complement cascade promotes the opsonization and destruction of pathogens, amplifying the inflammatory response; DAMP interactions involve mitochondrial or cytosolic DNA recognised by intracellular sensors, leading to inflammatory activation; and neutrophil degranulation releases antimicrobial factors and extracellular networks (NETs) to contain infection. (**B**): Hemostasis and platelet adhesion (right). This schematic illustrates platelet adhesion and clot formation in response to endothelial damage. Platelet adhesion: Platelets adhere to exposed collagen via receptors such as GPIb and GPIIb/IIIa, becoming activated. Platelet aggregation releases activating factors (e.g., ADP, thromboxane A2) that amplify platelet recruitment. Fibrin formation: thrombin converts fibrinogen to fibrin, stabilising the clot. Cell–vessel wall interactions: leukocytes adhere to endothelium via adhesion molecules such as selectins and integrins, contributing to inflammation.

The most significant processes are the following (Table 3 and Figure 4):

− Negative regulation of fibrinolysis (GO: 0051918)—Relevant proteins: A2M and PLG. Imbalances in this process may be associated with thrombosis or bleeding disorders. In autoimmune diseases, altered fibrinolysis may contribute to chronic inflammation.
− Complement activation (GO: 0006956)—Relevant proteins: C4A, C2, and other components of the complement cascade. This pathway plays a key role in systemic autoimmune diseases. Its excessive activation can damage tissue and perpetuate inflammation.
− Positive regulation of wound healing (GO: 0090303)—Relevant proteins: SERPINF1 and PLG. Critical for the resolution of inflammation and tissue remodelling. Deregulation could lead to fibrosis or repair defects.
− Regulation of response to stimuli (GO: 0032102 and GO: 0048584)—Relevant proteins: A2M, SERPINF1, and C9. These proteins are involved in the modulation of inflammation. Changes in their function are associated with uncontrolled immune responses.
− Coagulation and negative regulation of coagulation (GO: 0030195)—Relevant proteins: PLG and CPB2. Alterations in this balance contribute to thrombotic phenomena and cardiovascular disease.

**Table 3:** Enrichment analysis.

GO-Term	Description	Count in Network	Strength	Signal	False Discovery Rate
GO-0051918	Negative regulation of fibrinolysis	3 of 13	2.62	1.9	0.00032
GO-0006956	Complement activation	4 of 60	2.08	1.78	0.00032
GO-0090303	Positive regulation of wound healing	4 of 60	2.08	1.78	0.00032
GO-0032102	Negative regulation of response to external stimulus	6 of 387	1.44	1.38	0.00032
GO-0032101	Regulation of response to external stimulus	7 of 964	1.11	1.04	0.00032
GO-0080134	Regulation of response to stress	8 of 1373	1.02	0.93	0.00032
GO-0048584	Positive regulation of response to stimulus	9 of 2131	0.88	0.78	0.00032
GO-0048583	Regulation of response to stimulus	11 of 3931	0.7	0.59	0.00032
GO-0045861	Negative regulation of proteolysis	5 of 339	1.42	1.25	0.00082
GO-0006958	Complement activation, classical pathway	3 of 40	2.13	1.47	0.0015
GO-0006955	Immune response	7 of 1321	0.98	0.8	0.0015
GO-0030195	Negative regulation of blood coagulation	3 of 46	2.07	1.42	0.0018
GO-0002376	Immune system process	8 of 2121	0.83	0.66	0.0018

## 4. Discussion

SSc is a chronic, multisystem autoimmune disease characterised by cutaneous and visceral fibrosis, vasculopathy, and immune system changes. This results in a variety of changes in the molecular mechanisms underlying SSc, which are multifactorial and not yet fully understood in terms of how they interact to develop the different symptoms in these patients. The disease begins with microvascular injury, leading to endothelial cell dysfunction and profound molecular changes in the vascular system, such as endothelin-1 signalling [27].

SSc is also associated with a strong, dysregulated autoimmune response, including the production of specific autoantibodies (e.g., anti-topoisomerase I, anti-centromere, and anti-RNA polymerase III antibodies) or chronic inflammation with the high production of profibrotic cytokines (TGF-b, IL-3, IL-4, IL-6 and IL21) [28,29,30].

The present study focuses on analysing the proteomic differences between patients with systemic sclerosis (SSc), where we compared SSc patients exposed to silica dust with those not exposed. This research provides important information on the molecular mechanisms and pathogenic pathways involved, especially considering that silica exposure is associated with a higher prevalence of severe manifestations of the disease, such as diffuse cutaneous disease and interstitial lung disease. The findings add to the knowledge of the heterogeneity of SSc and how environmental factors influence its development and phenotypic expression.

### 4.1. Differential Proteins

This study identified eight proteins that were differentially regulated between the groups analysed. These proteins, all of which were reduced in the silica-exposed group, include adiponectin, immunoglobulins (IGLV3-19 and IGLV2-18), complement C2, alpha-2-macroglobulin (A2M), vitronectin (VTN), cytoplasmic actin 2, and pigment epithelium-derived factor (PEDF). Each of these molecules is involved in biological processes relevant to the pathogenesis of SSc, highlighting their potential contribution to the more severe phenotype observed in exposed patients.

- Adiponectin:

Its low expression in patients exposed to silica may contribute to endothelial dysfunction, chronic inflammation, and fibrosis, which are central processes in SSc. This protein has anti-inflammatory, anti-fibrotic, and vasculoprotective properties, which may be protective mechanisms lacking in the exposed group. Skin biopsies from patients with SSc have shown a reduction in the number and size of adipocytes (a major source of adiponectin) and their replacement by a fibrous matrix. In addition, lower levels of adiponectin have been reported in patients with SSc, both in the circulation and in skin, lung, and gastroscopic biopsies, compared to healthy controls, and significantly lower serum concentrations have been reported in patients with dcSSc compared to lcSSc [31,32,33]. Finally, an inverse correlation has been described between adiponectin levels in patients with SSc and the mRSS index, disease activity and progression, and a higher prevalence of scarring and pulmonary fibrosis [34,35].

- Immunoglobulin lambda variable 3–19 (IGLV3-19) and immunoglobulin lambda variable 2–18 (IGLV2-18):

This is a region of the variable domain of immunoglobulin light chains. Changwan Ryu et al. identified a proteomic profile in plasma samples from patients with SSc-ILD associated with platelet activation, cell adhesion, and immune responses, with IGLV3-19 being one of the thirty-eight proteins whose levels differed from healthy controls (upregulated, log 2-fold change 1.90) [36]. A positive correlation of IGLV3-19 with activated T cells and memory B cells has been reported in patients with SLE [37], which could imply its participation in immune hyperactivation and the production of autoantibodies, which could also be extended to other autoimmune diseases such as systemic sclerosis. Specifically, in this study, we describe IGLV-19 level differences between SSc patients exposed to silica and those not exposed. IGLV2-18, another variable domain region of immunoglobulin light chains, has never been reported as a differential marker in patients with SSc.

- Complement C2:

Reduced complement C2 may reflect the activation-associated consumption of the complement system, a common finding in autoimmune diseases. This activation perpetuates inflammation and tissue damage, which is likely to be exacerbated by exposure to toxicants. C2 deficiency has been linked to certain autoimmune diseases. In particular, complete genetic deficiency of complement component C2 is a strong risk factor for monogenic systemic lupus erythematosus (SLE), and partial deficiencies of C2 and C4A are important risk factors for SLE and primary Sjögren’s syndrome [27]. In a small study of 40 patients with scleroderma, Venneker et al. found that the incidence of partial C2 deficiency was higher than expected in patients with SSc [28]. On the other hand, complement activation is partly responsible for the tissue damage observed in autoimmune patients. Therefore, hypocomplementemia is a characteristic finding in patients with SLE, Sjögren’s syndrome, or antiphospholipid syndrome with active disease. However, in SSc, complement depletion is a less common finding, accounting for 15% of series, has been associated with the SSc activity index, functional disability, and the severity of general, cutaneous, vascular, cardiac, and pulmonary manifestations [29], and may be used to identify a specific subgroup of SSc patients with other overlapping connective tissue diseases [30].

- α2-macroglobulin (A2M) and vitronectin (VTN):

These proteins involved in coagulation and fibrinolysis are downregulated in exposed patients. Birkenmeier et al. [38] showed that patients with progressive systemic sclerosis have a state of hypercoagulation and hypofibrinolysis, suggesting that platelet and vascular dysfunction may play a key role in the pathogenesis of the disease, contributing to the characteristic microangiopathy. Our results are consistent with this hypothesis, since A2M could play a role in inhibiting plasmin (inhibiting fibrinolysis), thus contributing to the hypercoagulable state characteristic of the disease, and vitronectin could be related to the increase in fibrosis and endothelial dysfunction. On the other hand, in the study by Gundogdu et al. [39], serum levels of VTN were found to be lower in the SSc group compared to patients with SLE or healthy controls, suggesting the accumulation of VTN in fibrotic skin and subcutaneous tissues as a cause, which has already been demonstrated in experimental models of renal fibrosis [40].

- Actin, cytoplasmic 2:

Cytoplasmic actin 2, or gamma actin, is a protein that is widely expressed in the cellular cytoskeleton of muscle cells. Fibrosis is the hallmark of SSc and results from the excessive accumulation of the extracellular matrix in the skin and internal organs. This is associated with tissue damage, and one effect of this damage is the release of cytoskeletal proteins such as actin. Kimberly Showalter [41] mentions smooth muscle actin as a key marker in the cutaneous histology of SSc, as it is a protein expressed by myofibroblasts, cells involved in the cutaneous fibrosis characteristic of the disease, and it has been found that α-SMA levels in skin biopsies correlate with the severity of systemic sclerosis and with the response to treatment in clinical trials of antifibrotic drugs.

- Pigment epithelium-derived factor (PEDF):

This is a potent inhibitor of angiogenesis. Proteomic studies have identified PEDF as one of the most abundant proteins secreted by SSc skin fibroblasts compared to healthy controls [42]. A recent study showed that dermal fibroblasts in SSc play a direct role in the impairment of angiogenesis through the secretion of PEDF [43]. TGF-β signalling in fibroblasts was found to suppress angiogenesis via the secretion of PEDF, and this pathway remains active in explanted SSc skin fibroblasts. However, no increase in serum PEDF levels was found in dcSSc patients compared to healthy controls, consistent with the known paracrine mode of action of PEDF. Interestingly, PEDF has been shown to have an anti-fibrotic effect in a chemically induced liver fibrosis model, so it cannot be excluded that the TGF-β-induced PEDF expression observed in both SSc and idiopathic pulmonary fibrosis may represent an attempt to negatively feedback the fibrotic process [44].

### 4.2. Biological Pathway Analysis

Enrichment analysis highlights key processes such as the downregulation of fibrinolysis, complement activation, and immune response (Table 3 and Figure 4). These pathways reinforce the idea that silica exposure may not only act as a trigger for the disease but can also shape its clinical course by activating specific pathways that contribute to a more aggressive phenotype.

- Regulation of fibrinolysis and coagulation: Imbalances in these pathways may explain the thrombotic and vascular events observed in patients with SSc, particularly those with silica exposure.
- Complement activation: This key pathogenic mechanism in autoimmunity is relevant in the exposed subgroup, possibly associated with exacerbated inflammation and more pronounced tissue damage.
- Regulation of wound healing: Proteins related to this process, such as SERPINF1, suggest altered tissue remodelling favouring fibrosis, a characteristic finding in SSc.

### 4.3. Clinical Relevance

Treatment for SSc is multidisciplinary and focuses on controlling symptoms, preventing complications and delaying disease progression. As the disease develops for different reasons, treatment and even the response to drugs can vary. This work highlights the importance of identifying proteomic biomarkers that allow patients to be stratified into phenotypic subgroups, which could facilitate the development of targeted therapies. GLV3-19, associated with B-cell activation and autoantibody production, could be targeted through monoclonal antibodies (e.g., belimumab) to reduce immune hyperactivity. Complement C2 modulation via complement inhibitors (e.g., eculizumab) may help control inflammation and tissue damage. Adiponectin, with its anti-inflammatory and antifibrotic properties, could be therapeutically increased through receptor agonists or gene therapy. Alpha-2-macroglobulin (A2M) and vitronectin (VTN), both involved in coagulation and fibrosis, represent targets for thrombotic and vascular complications in autoimmune diseases. Finally, the pigment epithelium-derived factor (PEDF), implicated in angiogenesis regulation, could be modulated to improve vascular dysfunction in SSc.

Similarly, a blood-based biomarker panel measuring IGLV3-19, complement C2, adiponectin, A2M, VTN, and PEDF could aid in early disease detection and patient stratification. Prognostically, these markers could help predict disease progression and complications, such as fibrosis or vascular dysfunction. Additionally, they could be used to monitor treatment response, adjusting therapies in real-time based on biomarker levels, such as IGLV3-19 reduction after B-cell depletion therapy or adiponectin increases in antifibrotic treatment.

### 4.4. Limitations and Future Directions

Although this study provides a solid basis for investigating the effects of silica exposure on SSc, the relatively small sample size limits the generalisability of the results. Future research should include larger cohorts and assess the longitudinal effects of silica exposure on disease progression. In addition, functional studies investigating the specific roles of the identified proteins in SSc pathogenesis will be crucial to confirm their biological and clinical relevance.

To overcome the limitations of the current study, future research should focus on larger, multicentre studies to enhance the statistical power and generalizability of the findings. Expanding the sample size would allow for the better differentiation of disease subtypes and reduce biases introduced by small cohorts. Multicentric studies involving diverse populations would help validate the identified protein biomarkers across different genetic backgrounds and environmental exposures, ensuring their clinical relevance and reproducibility.

Additionally, a functional validation of the identified proteins is crucial to confirm their role in disease mechanisms and their potential as therapeutic targets. This could be achieved through in vitro and in vivo studies, including the following:

− Cell culture experiments to analyse how these proteins influence immune cell activation, fibrosis, and vascular dysfunction.
− Animal models of SSc to determine the impact of modulating these proteins on disease progression.
− CRISPR-based gene editing or RNA interference (siRNA/shRNA) to assess the direct effects of silencing or overexpressing these biomarkers in relevant cell types.
− Longitudinal patient studies to correlate protein expression levels with disease activity and treatment response over time.

## 5. Conclusions

This analysis reinforces the idea that silica exposure not only increases the risk of developing systemic sclerosis but also modulates its progression by altering pathways related to fibrinolysis, inflammation, coagulation, and fibrosis. In the words of Dr Steen, scleroderma is the disease of many faces [45]. Underlying this phenotypic variability may be different pathogenic pathways triggered by different external factors, many of which are toxic. Studying these interactions between environmental factors and molecular biology may open new doors to prognostic factors that will help in disease monitoring and more effective and personalised therapies.

## Figures and Tables

**Table 1:** List of exposed and nonexposed patients with their professions.

	Age *, Sex	Professions
Exposed		
si-SSC 1	52, F	Paper factory, production of dental prostheses
si-SSC 2	42, M	Marble
si-SSC 3	50, F	Agriculture
si-SSC 4	51, F	Foundry
si-SSC 5	35, M	Marble
si-SSC 6	80, F	Agriculture
si-SSC 7	60, F	Stone mill
si-SSC 8	45, M	Stonework
si-SSC 9	55, F	Agriculture
si-SSC 10	64, M	Foundry
si-SSC 11	74, M	Stonework
Nonexposed		
nosi-SSc 1	11, F	Housework
nosi-SSc 2	47, F	Administration
nosi-SSc 3	10, F	Cleaning
nosi-SSc 4	32, M	Administration
nosi-SSc 5	45, F	Housework
nosi-SSc 6	54, F	Bakery
nosi-SSc 7	40, F	Textile factory
nosi-SSc 8	30, F	Quality technique
nosi-SSc 9	39, F	Plastic factory
nosi-SSc 10	51, F	Bakery
nosi-SSc 11	23, F	Housework
nosi-SSc 12	55, F	Textile factory
nosi-SSc 13	22, F	Cook
nosi-SSc 14	-, F	Seamstress
nosi-SSc 15	36, F	Gardening
nosi-SSc 16	65, F	Dry cleaner
nosi-SSc 17	46, M	Upholstery
nosi-SSc 18	37, F	Hotels, car polishing
nosi-SSc 19	67, F	Administration
nosi-SSc 20	60, F	Screw factory
nosi-SSc 21	51, F	Seamstress

* Age at which scleroderma symptoms began, in years; F: female; M: male; -: data not available.

**Table 2:** Differential proteins between si-SSc and nosi-SSc groups.

Name	*m/z*	Group	*p*-Value	Fold Change	Log (Fold Change)
Q15848	ADIPO_HUMAN	Adiponectin OS = Homo sapiens OX = 9606 GN = ADIPOQ PE = 1 SV = 1	0.01	0.57	−0.24
P01714	LV319_HUMAN	Immunoglobulin lambda variable 3–19 OS = Homo sapiens OX = 9606 GN = IGLV3-19 PE = 1 SV = 2	0.01	0.61	−0.21
P06681	CO2_HUMAN	Complement C2 OS = Homo sapiens OX = 9606 GN = C2 PE = 1 SV = 2	0.02	0.77	−0.10
P01023	A2MG_HUMAN	Alpha-2-macroglobulin OS = Homo sapiens OX = 9606 GN = A2M PE = 1 SV = 3	0.02	0.71	−0.14
P04004	VTNC_HUMAN	Vitronectin OS = Homo sapiens OX = 9606 GN = VTN PE = 1 SV = 1	0.03	0.56	−0.24
P63261	ACTG_HUMAN	Actin, cytoplasmic 2 OS = Homo sapiens OX = 9606 GN = ACTG1 PE = 1 SV = 1	0.03	0.74	−0.12
P36955	PEDF_HUMAN	Pigment epithelium-derived factor OS = Homo sapiens OX = 9606 GN = SERPINF1 PE = 1 SV = 4	0.04	0.80	−0.09
A0A075B6J9	LV218_HUMAN	Immunoglobulin lambda variable 2–18 OS = Homo sapiens OX = 9606 GN = IGLV2-18 PE = 3 SV = 2	0.05	0.42	−0.37

## Data Availability

Data available on request due to privacy restrictions.
